# A study to evaluate the safety of platelet-derived growth factor for treatment of osteochondral defects of the talus

**DOI:** 10.1007/s00167-015-3549-0

**Published:** 2015-03-22

**Authors:** Alastair Younger, Kevin Wing, Murray Penner, Mark Cresswell

**Affiliations:** 1 Department of Orthopedics, University of British Columbia, 560 – 1144 Burrard Street, Vancouver, BC V6Z 2A5 Canada; 2 Department of Orthopaedics, University of British Columbia, 1000 – 1200 Burrard Street, Vancouver, BC V6Z 2C7 Canada; 3 Department of Radiology, University of British Columbia, St. Paul’s Hospital - 1081 Burrard Street, Vancouver, BC V6Z 1Y6 Canada

**Keywords:** Osteochondral defect, OCD, Osteochondritis dissecans, Platelet-derived growth factor, PDGF, Bone regeneration, Arthroscopic debridement

## Abstract

**Purpose:**

An arthroscopic procedure for the treatment of osteochondral defects using platelet-derived growth factor (PDGF) carried out in a matrix of tricalcium phosphate was developed. This prospective, case-series-based study was designed to evaluate the safety and clinical utility of this procedure.

**Methods:**

Patients with an isolated osteochondral defect larger than 5 mm long, 3 mm wide, and 5 mm deep and smaller than 30 mm long, 25 mm wide, or 20 mm deep were considered for enrolment. Only patients with chronic lesions were enroled. Arthroscopic debridement was followed by the placement of recombinant human PDGF in a matrix of tricalcium phosphate. The Ankle Osteoarthritis Scale (AOS), visual analogue scale (VAS) for pain, and SF-36 questionnaires were administered at 0, 2, 6, 12, and 24 weeks. Magnetic resonance imaging (MRI) and computed tomography (CT) scans were taken before and after surgery.

**Results:**

Five patients were ultimately enroled in this proof-of-concept trial. All outcome measures demonstrated marked improvement from baseline to final follow-up: The mean weight bearing VAS pain score improved by 49 %, and the mean AOS functional score improved by 28 %. Bone healing was seen on CT, and reduction in oedema signal was seen on MRI.

**Conclusion:**

This new procedure may offer a promising alternative for the treatment of osteochondral defects. Further high-quality studies are needed to confirm these results and to analyse the long-term effects of the procedure. The clinical relevance of this study is that the procedure may provide a less invasive option with improved bone healing compared to standard techniques .

**Level of evidence:**

IV.

## Introduction

The initial surgical treatment for osteochondral defect (OCD) of the talus is usually arthroscopic debridement via curettage. However, many patients do not achieve adequate pain relief or restoration of mobility with debridement alone [34, 36]. In addition, even among those patients for whom debridement appears to be effective, symptoms often recur over time.

For these patients, standard treatments include osteoarticular autograft (or allograft) transfer (OATS) [18, 19], or autologous chondrocyte implantation (ACI) [2, 3, 14, 17, 25]. These open procedures are more invasive and often more expensive than arthroscopy and can result in considerable morbidity. In addition, debridement does not repair the underlying bone defect [15] and may thus allow continued progression of the lesion [36].

Researchers have sought treatment alternatives for OCD that could stabilise the interior of the lesion and prevent further collapse [1, 2, 7]. This report introduces a new arthroscopic procedure in which a volume-filling, osteoconductive material containing a diffusible growth factor is inserted into the OCD lesion to stimulate repair of the patient’s own subchondral bone. This growth factor, recombinant human platelet-derived growth factor (rhPDGF), has been found to promote healing; regeneration; and repair of bone, ligament, periodontal cementum, and other tissues [9, 22–24, 26–33, 35].

In this new procedure, arthroscopic debridement to stable, viable bone is followed by arthroscopic placement of rhPDGF mixed with an osteoconductive matrix of beta-tricalcium phosphate (β-TCP). The defect is then sealed with fibrin glue, and the patient is kept non-weight bearing for 6 weeks prior to being remobilised.

The primary objective of this case-series-based study was to evaluate the initial safety, efficacy, and clinical utility of rhPDGF/β-TCP as an arthroscopic treatment following debridement for OCD of the talus, over the course of an initial 6-month follow-up. The hypothesis was that the procedure would result in improved pain and functional scores for patients with and OCD. The clinical relevance of this study is that, if successful, the procedure could provide a less invasive option with improved healing compared to standard techniques.

## Materials and methods

This single-site, single-surgeon, prospective study, conducted with enrolment from February 2012 until November 2012, was designed as a case-series-based clinical study.

Patients diagnosed with a talar OCD were recruited for the study from the clinical practice of the authors. Inclusion criteria included signed REB-approved informed consent form (ICF) prior to enrolment; diagnosis of an isolated OCD larger than 5 mm long, 3 mm wide, and 5 mm deep, confirmed by MRI; independent, ambulatory and can comply with all post-operative evaluations and visits; ≥21 years of age or older; skeletally mature; stable ankle joint on history and similar ligament stability with the opposite ankle; <15° of hindfoot valgus and 5° of hindfoot varus; OCD is chronic and not secondary to acute trauma within the previous 6 months; if history of fracture, no residual deformity of the tibia, fibula, or syndesmosis; no prior fusions of the hindfoot (subtalar and talonavicular joints); body mass index (BMI) ≤35; American Society of Anesthesiologists (ASA) physical status classification of 1 or 2; and has exhausted non-operative treatment. Exclusion criteria included >15° of hindfoot valgus or 5° of hindfoot varus; defect >30 mm length, 25 mm width, or 20 mm depth in size on MRI assessment; allergy to yeast-derived products; has metallic or electronically, magnetically, or mechanically activated implants that would contraindicate MRI scans of the foot; history of malignancy anywhere in the body; physically or mentally compromised and unable or unlikely to remain compliant to follow-up; history of drug/alcohol abuse within the 12 months prior to screening for study entry; pregnant, or able to become pregnant but not practicing a medically accepted form of birth control; current acute infection in area surrounding surgical site; history of anaphylaxis; condition is bilateral and surgery is to be scheduled over the course of the study; requires concomitant osteotomy of tibia, fibula, or calcaneus for hindfoot deformity, or requires concomitant hindfoot fusion for hindfoot arthritis or deformity; undergoing any concomitant surgery that may invalidate outcome scores for this study; OCD of the tibia in isolation or in combination with the talar lesion; nicotine addiction or using medication or substances containing nicotine; cocaine abuse or use of cocaine derivatives; undergoing revision debridement of an OCD.

### Data collection

Diagnosis of OCD was confirmed via baseline magnetic resonance imaging (MRI) scans of the lesion site prior to enrolment. Location and size of defect (width, depth, and length), were determined via ankle arthroscopy and recorded prior to treatment. Photographs of defect before and after debridement as well as any associated pathology (osteophytes, synovitis) were also obtained.

Patient demographics, comorbidities, and diagnoses were recorded preoperatively. Patient assessments were completed preoperatively and at 2, 6, 12, and 24 weeks post-operatively. Clinical outcomes were recorded preoperatively and at each follow-up visit using the visual analogue scale (VAS) for pain [21], the Ankle Osteoarthritis Scale (AOS) [11], the short-form-36 (SF-36) Health Survey [6], and AP and lateral X-rays. All adverse events affecting the ankles were also recorded at each study visit (see “Appendix”).

Follow-up MRI was conducted at 12 and 24 weeks, in order to evaluate bone marrow oedema. In addition, CT scans at 2 and 24 weeks were compared in order to determine healing status at the base of the OCD and fill of the graft. All scans were measured using InteleViewer software tools (Intelerad, Calgary, AB, Canada), which enable automated, quantitative segmentation and calculation of two-dimensional area and three-dimensional volume measurements (with read-outs in 1-mm increments), as well as image registration for pre- and post-treatment comparisons of patient lesions. The MRI scans were evaluated with sagittal and coronal sequences cut with the greatest amount of oedema (T2-weighted signal on fat suppressed images) being measured for square surface area of involvement. The oedema signal was measured preoperatively and at each follow-up period. The total area in square millimetres was analysed using the analysis of variance. The CT scan was evaluated for size of defect preoperatively and for fill with bone graft at both time periods. The CT was measured on one 0.6-mm slice on the sagittal view and one 0.6-mm slice on the coronal view.

### Surgical techniques

All procedures were performed by a single surgeon having advanced skills in arthroscopy and foot and ankle surgery. Patients were treated with arthroscopic debridement of the OCD to stable, viable bone via abrasive osteochondroplasty without microfracture or drilling. (Microfracture was not used because the subchondral bone was breached and softened in all cases). Debridement was followed by arthroscopic placement of a preparation containing rhPDGF and β-TCP (see below).

Routine ankle arthroscopy was performed using anterior medial, anterior lateral, and medial portals to access and debride the joint. An Augment Bone Graft preparation (BioMimetic Inc., Memphis, TN) containing β-TCP and rhPDGF was then prepared using standard techniques and placed into the joint via the arthroscopic portal. Following mixing, the final consistency of the graft material is similar to that of granular wet sand. When placed near a viable host bone, it acts as scaffold for new bone growth; it subsequently undergoes remodelling and is finally replaced by host bone [8]. All materials used in the components have been previously approved by Health Canada for the treatment of foot and ankle fusion procedures and by the FDA for the treatment of periodontal bone defects.

Fibrin glue was then placed over the defect to ensure containment of TCP granules. The foot was dorsiflexed while the fibrin glue set, then the ankle kept dorsiflexed until the wounds were closed and the limb placed in a below-the-knee slab in dorsiflexion for 2 weeks post-surgery.

If all appeared stable at 2 weeks, subjects began joint range-of-motion exercises in a walker boot and remained non-weight bearing until the 6-week follow-up visit.

### Outcome measures

The primary outcome measures were the VAS for pain and the Ankle Osteoarthritis Scale (AOS). The VAS for pain is a unidimensional, self-reported measure of pain intensity [16, 20, 21], which has been widely used in diverse adult populations, including those with rheumatic disease [12, 13]. The AOS is a reliable, validated, patient-reported, ankle-specific functional outcome instrument [11]. Both the pain and disability components were used to calculate the total AOS score. (The score ranges from 0 to 100, with a lower score indicating more normal function). Secondary outcome measures included the physical component summary (PCS) and mental component summary (MCS) subscales of the SF-36 Health Survey questionnaire, and MRI and CT scans of the affected ankles. The SF-36 is a generic, patient-reported measure of general health status [4, 6] that has been shown to have acceptable criterion validity in patients with end-stage ankle arthritis [20].

This study was approved by the Institutional Review Board of the Providence Health Care Research Institute at the University of British Columbia (REB #H03-50062) and was therefore performed in accordance with the ethical standards laid down in the 1964 Declaration of Helsinki and its later amendments. All patients provided informed consent for study enrolment and for the surgical procedure prior to questionnaire administration.

### Statistical analysis

Treatment safety and efficacy were assessed by comparing the preoperative baseline scores on the primary and secondary outcome measures with the scores recorded during follow-up evaluations at 6, 12, and 24 weeks, using Student’s *t* test. Ninety-five per cent confidence intervals were used to assess variability using AOS scores and mean area of oedema (mm^2^) via MRI.

## Results

Thirty patients with verified OCD were initially assessed for inclusion in this pilot study. Of these, 25 patients were excluded due to ankle instability or other associated pathology, or were from the practice of another surgeon.

Five patients with six OCD lesions of the talus met all inclusion and exclusion criteria, and underwent arthroscopic debridement followed by arthroscopic filling of the defect with rhPDGF-BB/β-TCP matrix. None of these patients withdrew from the study before the final follow-up visit at 24 weeks; all patients were seen for follow-up at the correct intervals and had complete data.

Mean patient age was 52 ± 8.5 years, and mean BMI was 26.3 ± 5.0. Three of the patients were female and two were male. In one patient, the OCD occurred in the left ankle; in the remainder, it occurred in the right ankle. One patient had two lesions on the same ankle (one on the medial and one on the lateral side of the talus). Defect parameters are summarised in Table 1. Four patients had undergone a prior debridement, and three had undergone more than one prior debridement. Four lesions were shoulder lesions and two were not.

**Table 1:** Dimensions of OCD lesions from arthroscopic assessment

	Average	Standard deviation
Length (mm)	14	3
Width (mm)	9	2
Depth (mm)	11	2
Surface area^a^ (mm^2^)	103	37
Volume^b^ (mm^3^)	285	139

^a^Surface area was calculated as an ellipse

^b^Volume was calculated as a hemi-ellipsoid

**Fig. 1:**
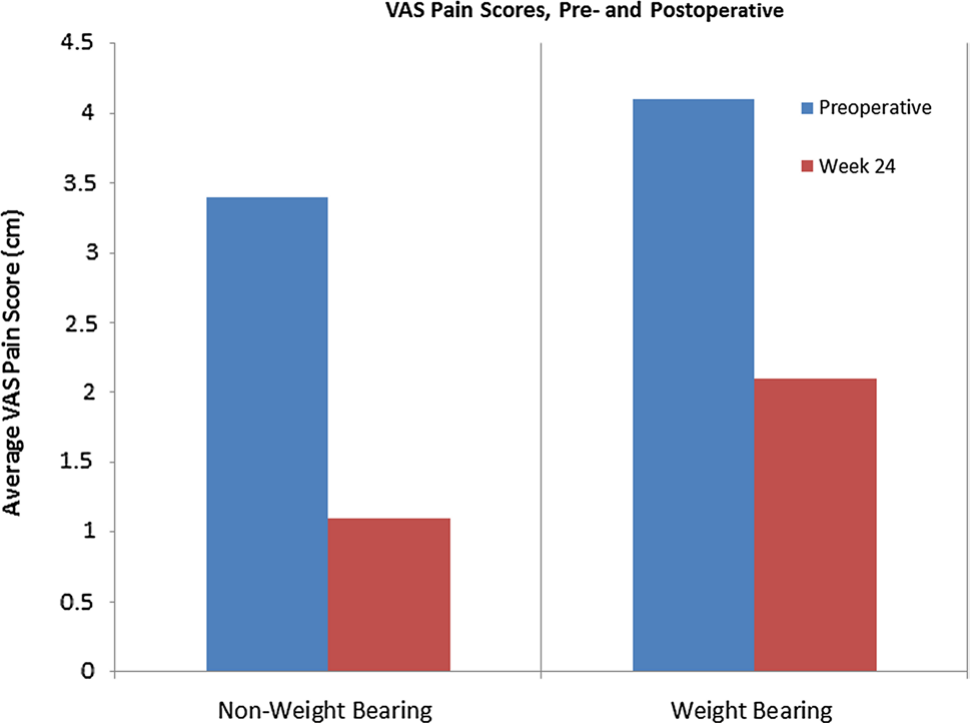
Average scores on the VAS pain questionnaire, from preoperative baseline (preop) to final follow-up (week 24). A lower score indicates decreased pain

**Fig. 2:**
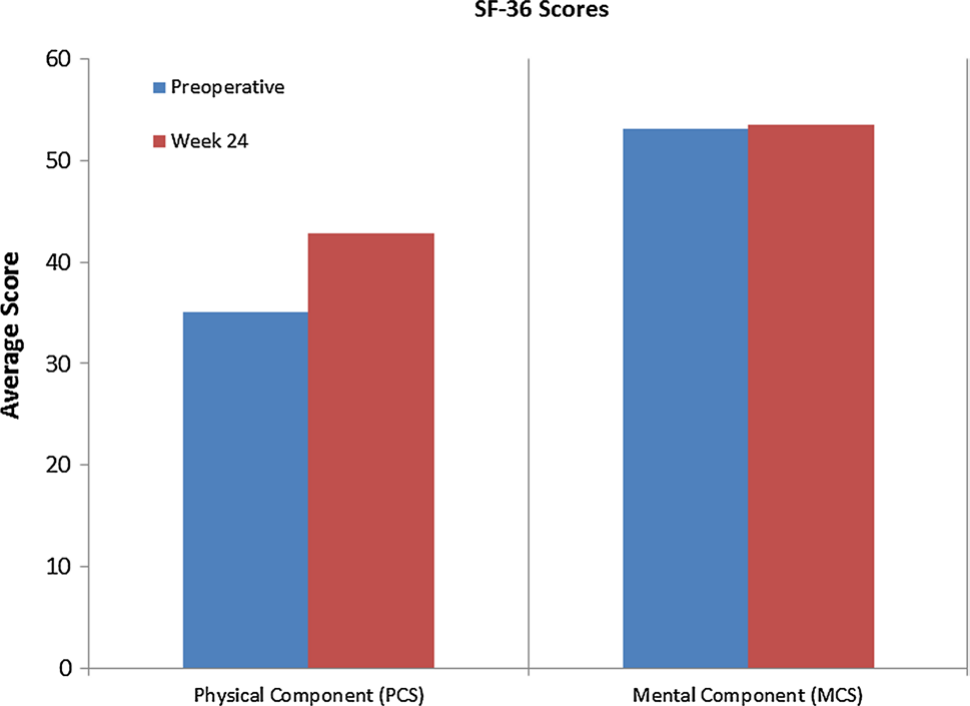
Average scores on the mental and physical components of the SF-36 Health Survey questionnaire, from preoperative baseline (preop) to final follow-up (week 24). Increased scores indicate improvement

**Fig. 3:**
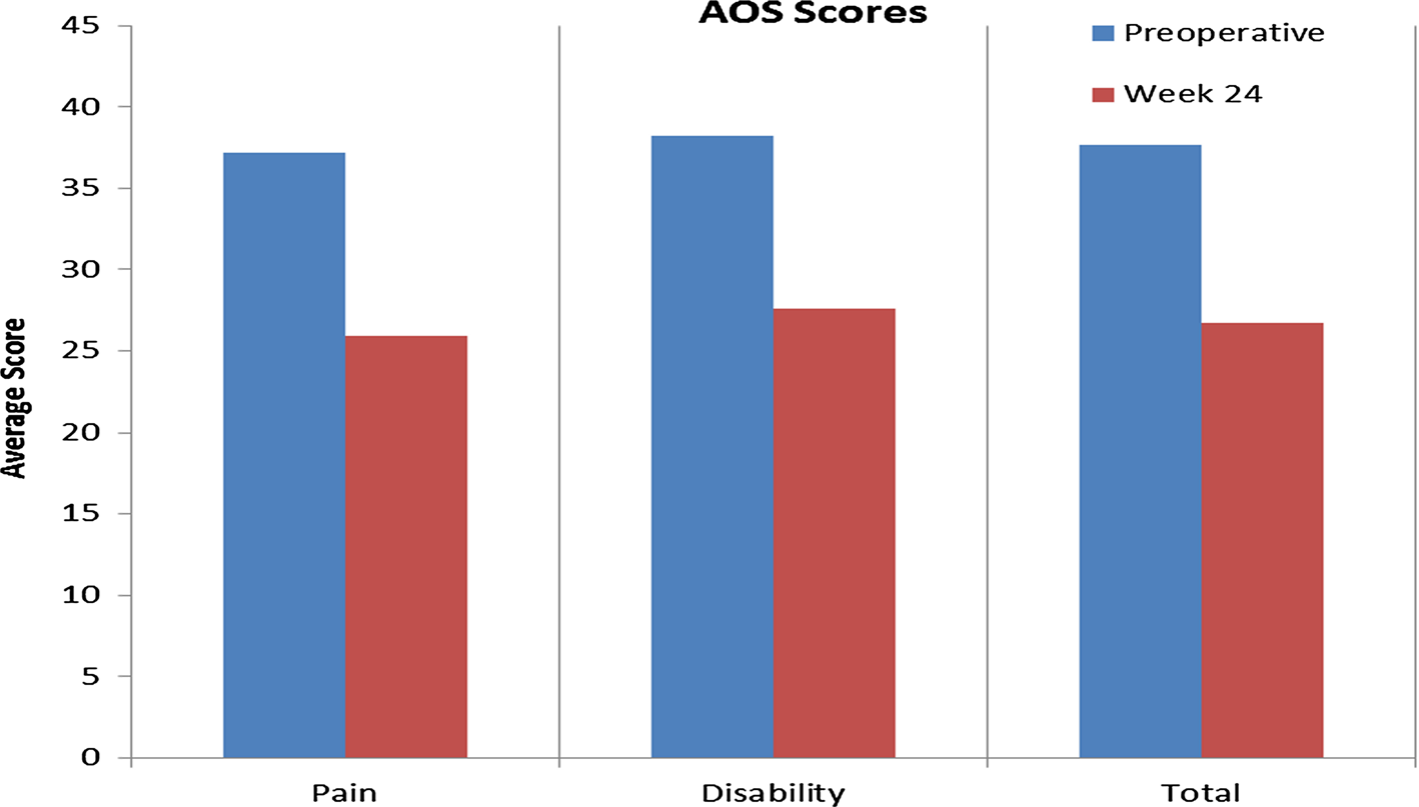
Average scores on the total Ankle Osteoarthritis Scale (AOS) and on the pain and disability components, from preoperative baseline (preop) to final follow-up (week 24). Decreased scores indicate improvement

Mean scores for pain and disability at baseline and at 24 weeks post-operative follow-up (as measured by the VAS, SF-36, and AOS scales) are listed in Table 2. Changes in these scores are illustrated in Figs. 1, 2 and 3.

**Table 2:** Mean outcome scores, pain, and disability

	VAS pain scale		AOS			SF-36	
	Non-WB	WB	Pain	Disability	Total	PCS	MCS
Preoperative baseline	3.4	4.1	37.2	38.2	37.7	35.1	53.2
Week 24	1.1	2.1	25.9	27.6	26.7	42.9	53.5

*VAS* visual analogue scale, AOS Ankle Osteoarthritis Scale, *SF-36* short-form (36) Health Survey, *WB* weight bearing, *PCS* physical component summary, *MCS* mental component summary

**Fig. 4:**
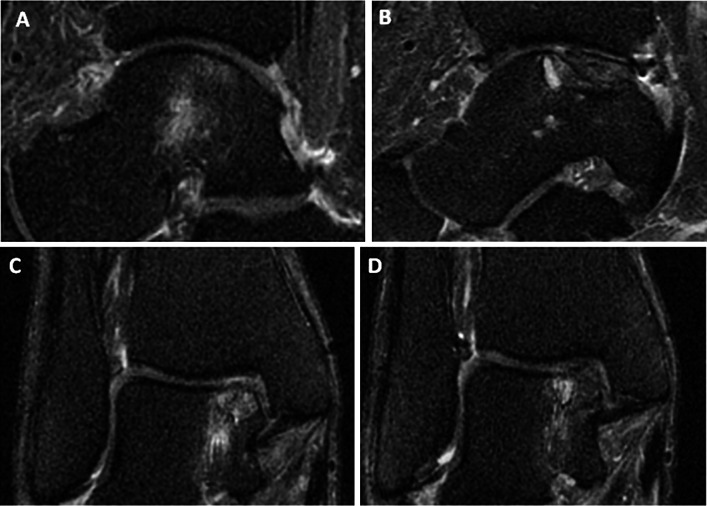
Magnetic resonance imaging (MRI) scans. **a** *Preoperative sagittal* view showing bone marrow oedema. **b** *Post-operative sagittal* view showing reduction in the oedema signal. **c** *Preoperative coronal* view showing bone marrow oedema. **d** *Post-operative coronal* view showing reduced marrow signal

No major adverse events related to the graft were observed. Over the course of the study, there were no wound infections, wound breakdowns, reoperations, loss of motion, symptoms of loose body/impingement, or synovitis. (Any additional adverse events affecting the ankle are listed by patient in the “Appendix”).

Evaluation of bone marrow oedema as visualised by MRI, preoperatively and at 24 weeks post-operatively, is shown in Table 3 and Fig. 4 (note that retrograde drilling was not possible in this joint, as only minimal cartilage remained).

**Table 3:** Mean area of oedema (mm^2^) via MRI

Parameter	Preop	24 weeks
Coronal oedema	95 (CI 20–171)	71 (CI 0–208)
Sagittal oedema	140 (CI 50–229)	37 (CI 0–93)
Average	118 (CI 45–190)	54 (CI 0–150)

**Table 4:** Mean area of OCD lesions and percentage filled by graft, as visualised via CT scan

Parameter	2 weeks	24 weeks
Coronal view (mm^2^)	69	58
Sagittal view (mm^2^)	89	76
Amount of defect filled by graft (coronal view) (%)	85.2	54
Amount of defect filled by graft (sagittal view) (%)	84.6	61

**Fig. 5:**
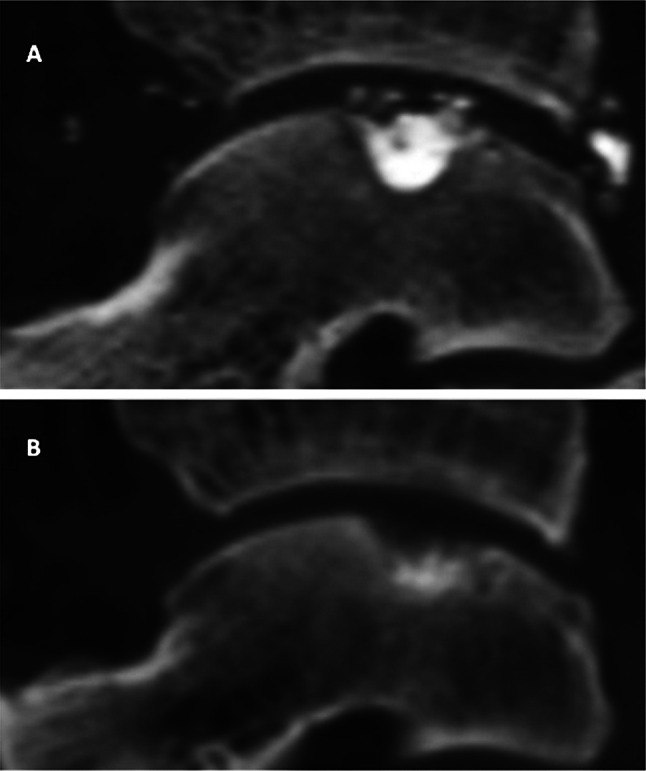
Post-operative computed tomography (CT) scans. **a** *Coronal* view at 2 weeks showing fill of the osteochondral defect. **b** *Coronal* view at final scan (24 weeks) showing healing of the bone in the base of the osteochondral defect and resorption of periarticular tricalcium phosphate granules

Incorporation of the graft as visualised by CT scan, at 2- and 24-week follow-ups, is shown in Table 4 and Fig. 5.

## Discussion

The most important finding of the present study was that a new arthroscopic procedure yielded improved pain and functional scores for patients with OCD, and enhanced healing of these lesions (as confirmed by MRI and CT scans). No short-term complications were observed despite close monitoring of the patients. This prospective, single-centre, case-series-based, proof-of-concept study evaluated the initial safety, efficacy, and clinical utility of rhPDGF/β-TCP as an arthroscopic treatment following debridement for OCD of the talus in five patients.

Results at 6-month follow-up indicate that the method is both safe and effective, at least initially. Mean non-weight bearing VAS pain scores decreased by 68 %, mean weight bearing VAS pain scores decreased by 49 % (VAS), and mean scores on the AOS disability component decreased by 28 % (Table 2; Figs. 1, 3). MRI scans confirmed a reduction in subchondral oedema over time in all patients, with the mean area decreasing by 25 % on the coronal view and by 74 % on the sagittal view from preoperative baseline to final follow-up scans at 24 weeks (Table 3; Fig. 4). No heterotopic ossification was evident on follow-up CT scans, and the graft was incorporated in all five cases (Table 4; Fig. 5). In addition, no major adverse events related to the graft were observed, and none of the patients required reoperation for their OCD.

With procedures such as OATS, not only is there increased risk of knee donor-site morbidity [19], but the force of impact during graft insertion can damage both the talus and the chondrocytes in the graft [5]. Thus, gentler insertion of the non-cellular preparation used in our procedure confers the added advantage of reduced damage to ankle and graft, as well as the knee.

In a large, multi-centre, prospective, randomised, controlled, clinical trial, DiGiovanni et al. [10] compared the safety and efficacy of PDGF/β-TCP versus autogenous bone grafts (autografts) in patients requiring hindfoot or ankle fusion. The study found that the PDGF treatment resulted in comparable fusion rates, less pain, and fewer side effects compared to treatment with autograft. Due to its role as a mitogenic and chemotactic factor for fibroblasts [30, 32], osteoblasts [22–24, 26, 29], chondrocytes [28, 33], and tenoblasts [9, 27, 35], PDGF also holds considerable promise in restoring bone integrity in lesions such as OCDs. Healing and restoration of the subchondral bone may thus make our method more effective in the long term than cartilage grafts, OATS, or ACI. In addition, this new procedure offers an alternative that should prove to be less invasive and more economical than either OATS or ACI.

The current study has several strengths. Its prospective design provides proof of concept in support of the original hypothesis. Especially promising was the fact that four of the five patients had undergone previous debridements that required revision, but no revisions or additional procedures were required following the rhPDGF/β-TCP treatment. Limitations included small sample size, a short follow-up period, lack of a concurrent control group, and potential selection bias due to patient recruitment from a single clinical centre. In addition, the improved area of marrow oedema observed at final follow-up could be due in part to a period of relatively limited mobility. A larger, multi-centre, randomised, controlled trial with a minimum 2-year follow-up period is needed and is planned in order to address these limitations.

The clinical relevance of this study is that it demonstrates that a new technique using a human growth factor for the treatment of OCD can be safe, well tolerated, and clinically useful. If the results of the present study are confirmed in a larger trial, the potential benefits include lower cost, less post-operative pain, fewer wound complications, and shorter recovery time due to its less invasive nature; no non-unions of osteotomies; considerable reductions in patient-reported pain several months after surgery; and better long-term outcomes, compared with more invasive procedures such as OATS or ACI, which are currently the standard of care. In addition, there is no need for grafts (and therefore no donor-site morbidity), and this mixture of growth factor and osteoconductive matrix may help to stabilise the lesion after debridement, preventing further progression.

## Conclusion

In conclusion, the use of rhPDGF in an osteoconductive matrix shows promise as a clinically useful alternative for the treatment of OCD lesions of the talus following arthroscopic debridement. An expanded series or randomised, controlled trial is required to confirm these results.

## Appendix: adverse events

See Tables 5, 6, and 7.

**Table 5:** Adverse events as reported by patients

Patient ID	Adverse event description	Onset date
001	Fell using crutches, stuck splint at study ankle	12 March 2012
	Slipped on stairs, muscles sore at ankle	11 May 2012
	Development of stress fracture at calcaneus of study foot (osteoporosis-Z-score-3.7, severely osteoporotic)	Noted during May 2012
	Small clot with superficial phlebitis right ankle	Noted during 17 May 2012 appointment
	Severed ACL right leg (study side)	Noted during unscheduled visit 18 July 2012
	Fell off bike, landed on right side (knee area)	Early August 2012
	Occasional catching and locking symptoms study ankle	Noted during 24 August 2012 visit
	Medial pain, study ankle	Noted during 24 August 2012 visit
	Dropped 1-lb weight on study ankle	Noted during 24 August 2012 visit
002	Right hip replacement	6 March 2013
	Continued pain left study ankle. Bony prominence subtalar joint noted during May 9 follow-up—patient had injection for this	Noted during unscheduled visit 4 April 2013
003	Cast causing pain—removed and replaced	Noted during unscheduled visit 23 October 2012
	Sharp, acute pain right study ankle	5 November 2012
	Shooting pains medial ankle, decreased sensation throughout entire dorsum of foot	Noted during 15 November 2012 visit
	Patient notes colour difference right study ankle, swelling in foot, lump on bottom of foot (noticed during December 2012), sore big toe with pain radiating to foot, numbness	Noted during unscheduled visit 21 December 2012
	Severe OA left knee—referred to specialist	Referral 27 December 2012
	After short walk, sharp pain in ankle	6 January 2013
	Corneal surgery for displaced lens	10 January 2013
004	Graft not contained as well as hoped	Noted during 7 June 2012 visit
	Stiff ankle	Noted during 12 July 2012
	Ankle becomes swollen after running any distance	Noted during 15 November 2012 visit
005	Slipped in elevator, caught himself	24 June 2012
	Patient notes occasional numb/tingling sensation	Noted during 25 June 2012 visit
	Residual material back of joint	Noted during 25 June 2012 visit
	Dropped brick on ankle	16 August 2012
	Withdrawal symptoms from discontinuation of tramadol—insomnia, loss of appetite	Noted during 30 August 2012 visit

**Table 6:** Adverse event definitions: severity criteria

Degree	Description
Mild (Grade 1)	Symptom(s) barely noticeable to subject or does not make subject uncomfortable; does not influence performance or functioning; prescription drug not ordinarily needed for relief of symptom(s), but may be given because of the nature of subject
Moderate (Grade 2)	Symptom(s) of a sufficient severity to make subject uncomfortable; performance of daily activity is influenced; subject is able to continue in study; treatment of symptom(s) may be needed
Severe (Grade 3)	Cause severe discomfort; symptoms cause incapacitation or significant impact on subject’s daily life; severity may cause cessation of treatment with study device; treatment for symptom(s) may be given and/or subject hospitalised
Life-threatening (Grade 4)	Extreme limitation in activity; significant assistance required; significant medical intervention/therapy required; hospitalisation or hospice care required

**Table 7:** Adverse event definitions: relationship to procedure

Relationship	Description
Not related	Any reaction that does not follow a reasonable temporal sequence from administration of the study device AND that is likely to have been produced by the subject’s clinical state or other modes of therapy administered to the subject
Unlikely	Any reaction that does not follow a reasonable temporal sequence from administration of the study device or that is likely to have been produced by the subject’s clinical state or other modes of therapy administered to the subject
Likely	A reaction that follows a reasonable temporal sequence from administration of the study device OR that follows a known response pattern to the suspended device AND that could not be reasonably explained by the known characteristics of the subject’s clinical state or other modes of therapy administered to the subject
Definite	A reaction that follows a reasonable temporal sequence from administration of the study device AND that follows a known response pattern to the suspected device AND that recurs with rechallenge, and/or is improved by removing the device
